# Pharmacophore Modeling and Molecular Docking Studies on *Pinus roxburghii* as a Target for Diabetes Mellitus

**DOI:** 10.1155/2014/903246

**Published:** 2014-07-10

**Authors:** Pawan Kaushik, Sukhbir Lal Khokra, A. C. Rana, Dhirender Kaushik

**Affiliations:** Institute of Pharmaceutical Sciences, Kurukshetra University, Kurukshetra 136119, India

## Abstract

The present study attempts to establish a relationship between ethnopharmacological claims and bioactive constituents present in *Pinus roxburghii* against all possible targets for diabetes through molecular docking and to develop a pharmacophore model for the active target. The process of molecular docking involves study of different bonding modes of one ligand with active cavities of target receptors protein tyrosine phosphatase 1-beta (PTP-1*β*), dipeptidyl peptidase-IV (DPP-IV), aldose reductase (AR), and insulin receptor (IR) with help of docking software Molegro virtual docker (MVD). From the results of docking score values on different receptors for antidiabetic activity, it is observed that constituents, namely, secoisoresinol, pinoresinol, and cedeodarin, showed the best docking results on almost all the receptors, while the most significant results were observed on AR. Then, LigandScout was applied to develop a pharmacophore model for active target. LigandScout revealed that 2 hydrogen bond donors pointing towards Tyr 48 and His 110 are a major requirement of the pharmacophore generated. In our molecular docking studies, the active constituent, secoisoresinol, has also shown hydrogen bonding with His 110 residue which is a part of the pharmacophore. The docking results have given better insights into the development of better aldose reductase inhibitor so as to treat diabetes related secondary complications.

## 1. Introduction 

Diabetes mellitus is one of the very common chronic diseases across the world and the number of diabetic patients is on the rise. The World Health Organization (WHO) estimates that about 200 million people all over the globe are suffering from diabetes and this figure is likely to be doubled by 2030. WHO says that about 80% of the deaths occur every year due to diabetes in middle-income countries [1]. The recently published Indian council for medical research-India diabetes (ICMR-INDIAB) national study reported that there are 62.4 million people with type 2 diabetes (T2DM) and 77 million people with prediabetes in India [2]. This will be increased to 100 million by 2030 [3]. T2DM predominantly affects older individuals in developed countries, while in developing nations like India, it is affecting the younger population in the prime of their working lives and thus poses an even greater threat to the health of these individuals [2, 4]. Many Indian medicinal plants are being examined for their beneficial use in diabetes and reports on merits of using such plants occur in numerous scientific journals. Bark of many plants along with* Pinus roxburghii* has been used to treat diabetes ethnopharmacologically [5].* Pinus roxburghii *is known to be a rich source of terpenoids, flavonoids, tannins, xanthones, steroids [6, 7], and so forth. There is much interest among the scientist to use this for therapeutic purposes.* Pinus roxburghii* is also attributed to many pharmacological activities like analgesic, anti-inflammatory [8], anticonvulsant [9], antiasthmatic [10], hepatoprotective [11], and antidyslipidemic [12].

The receptor targets for T2DM reported by many scientists till date are glycogen phosphorylase, protein tyrosine phosphatase 1-beta (PTP-1*β*), dipeptidyl peptidase-IV (DPP-IV), glucokinase, peroxisome proliferator activated receptor (PPAR-*γ*), aldose reductase (AR), insulin receptor (IR), and so forth [13]. Protein tyrosine phosphatases are a group of enzymes that remove phosphate groups from phosphorylated tyrosine residues in proteins. A large body of evidence from a variety of experimental systems has strongly implicated a key role for PTP1*β* in the regulation of the insulin signaling pathway [14]. The insulin receptor (IR) is a transmembrane receptor that is activated by insulin, IGF-I (insulin-like growth factor I), IGF-II (insulin-like growth factor II) and belongs to the large class of tyrosine kinase receptors [15]. Metabolically, the insulin receptor plays a key role in the regulation of glucose homeostasis, a practical process that under deteriorate conditions may result in a range of clinical manifestations including diabetes and cancer [16, 17]. Binding of insulin leads to phosphorylation of several intracellular substrates, including insulin receptor substrates (IRS1, 2, 3, 4), Casitas B-lineage (CBL), and other signaling intermediates [18]. Dipeptidyl peptidase-IV (DPP-IV), also known as adenosine deaminase complexing protein, is a protein that, in humans, is encoded by the* DPP4* gene [19]. Inhibition of DPP-IV has been shown to be an appropriate treatment for T2DM [20]. DPP-IV specifically removes N-terminal dipeptides from substrates containing proline or alanine as the second residue, transforming them into inactive or even antagonistic species. The most imperative DPP-IV substrates are incretins, such as glucagon-like peptide-1 (GLP-1) and glucose dependent insulinotropic polypeptide (GIP), which stimulates insulin secretion [21]. Aldose reductase (AR) is the first enzyme of the polyol pathway and is widely distributed in mammalian tissues. Due to increased aldose reductase activity, the accumulation of intracellular sorbitol is also raised. It implicates the development of various secondary complications of diabetes mellitus [22].

The main objective of this study is to validate the ethnopharmacological knowledge of* Pinus roxburghii* with the help of modern computer aided drug designing tools and to develop safe and more reliable treatment for diabetes.

## 2. Material and Methodology

### 2.1. Receptor

The three-dimensional crystal structure of different receptors taken from Protein Data Bank (PDB) (http://www.rcsb.org/) is as follows: IR (PDB ID: 1IR3), AR (PDB ID: 1US0), PTP1*β* (PDB ID: 2F70), and DPP-IV (PDB ID: 3F8S) [23]. All the PDB's were loaded in the Molegro virtual docker (MVD) with the removal of all water molecules. The standard Molegro algorithm was utilized for rendering the missing charges, protonation states, and assigning of polar hydrogen to the receptor.

### 2.2. Ligands

The mol files and smile formula of ligands were obtained from CHEMSPIDER database [24]. Structures of ligands were drawn using marvin sketch and energy minimization was done using MMFF94 force field. Energy minimization is done to help the docking programme for identifying the bioactive conformer from the local minima. One major advantage of MVD is that it helps in assigning the missing bond orders, charges, bonds, and hybridization states of the imported ligands. The 2D structures of 25 ligands are illustrated in Table 1.

### 2.3. Molinspiration

Molinspiration, an online tool, was employed to perform QSAR studies in order to identify potential activators of biological objects. It offers free online services for calculation of important molecular properties (LogP, polar surface area, number of hydrogen bond donors, and acceptors), as well as prediction of the bioactivity score for the most important drug targets. Molinspiration tool was used to compute properties of ligands such as molecular weight, logP, H bond acceptors, and H bond donors [25]. These filters help in early preclinical development and could help in avoiding costly late step preclinical and clinical failure. Lipinski's rule of five was applied to select probable ligands [26]. The constituent that had more than one violation was eliminated from the present study.

### 2.4. Validation and Analysis of Docked Receptor-Ligand Complex Structures

To ensure that ligands docked using the Molegro virtual docker represent valid score and accurate binding with receptor, the MVD scoring algorithm was to be validated first for the crystal structures (PDB: 1IR3, 1USO, 2F70, 3F8S). In view of this, IR (PDB ID: 1IR3) was tasted again with molecule: ANP (phosphoaminophosphonic acid-adenylate ester); AR (PDB ID: 1USO) was tested with IDD594 (2-(4-bromo-2-fluoro-benzylthiocarbamoyl)-5-(fluoro-phenoxy)-acetic acid); PTP1*β* (PDB ID: 2F70) was tested with UN608(3-{[3-(3-sulfoamino-phenyl)-propionylamino]-methyl}-phenyl)-sulfamic acid; DPP-IV (PDB ID: 3F8S) was tested with PF2 (2-(4-{(3S,5S)-5-[(3,3-difluoropyrrolidin-1yl)carbonyl]pyrrolidin-3-yl}piperazin-1-yl)pyrimidine). They served as control docking models as illustrated in Table 2. The outcome of the docking showed that MVD determined the optimal orientation of the internal ligands.

### 2.5. Molecular Docking of Ligands

We used MVD, which has been recently introduced and gained attention among medicinal chemists [27]. Bench mark results of MVD software provide very accurate predictions of ligand binding modes (87.0%) compared with other docking software such as Glide (81.8%), GOLD (78.2%), Surflex (75.3%), and FlexX2 (57.9%) [28]. MVD is based on a differential evolution algorithm called MolDock; MolDock Score energy, *E*_score_, is defined by (1), where *E*_inter_ is the ligand-receptor interaction energy and *E*_intra_ is the internal energy of the ligand. *E*_inter_ is calculated according to (2):

(1) $$\begin{array}{c} E_{\text{score}}=E_{\text{inter}}+E_{\text{intra}}, \end{array}$$

(2) $$\begin{array}{c} E_{\text{inter}}=\;\underset{i=\text{ligand}}{\sum }\;\underset{j=\text{protein}}{\sum }\left[E_{\text{PLP}}\left(r_{ij}\right)+332.0\frac{q_{i}q_{j}}{4r_{ij}^{2}}\right]. \end{array}$$

The *E*_PLP_ term is a “piecewise linear potential” [29, 30] that uses two different parameters, one for the estimate of the steric term (van der Waals) between atoms and another for the potential for hydrogen bonds; it describes the electrostatic interactions between charged atoms [28]. *E*_intra_ is calculated according to (3).

(3) Eintra=  ∑i=ligand ∑j=protein[EPLP(rij)]+∑flexible  bondA[1−cos⁡⁡(mθ−θ°)]+Eclash.

The first term in (3) calculates all the energies involving pairs of atoms of the ligand, except those associated with two bonds. The second term represents the torsional energy, where *h* is the torsional angle of the bond. The average of the torsional energy bond contributions is used if several torsions can be determined. The last term, *E*_clash_, assigns a penalty of 1,000 kcal/mol if the distance between two heavy atoms (more than two bonds apart) is smaller than 2.0 Å, ignoring infeasible ligand conformations [28].

The molecular docking was performed for all the constituents with the predicted cavities of the receptor. The MolDock score (GRID) function was used with a grid resolution (Å) of 0.30 and a binding site radius of 12 Å with respect to the origin of the respective cavities. The “MolDock SE” searching algorithm 10 runs using a maximum of 1500 iterations with a total population size of 50 was applied. The energy threshold used for the minimized final orientation is 100. The simplex evaluation with 300 maximum steps of neighbor distance factor 1 was completed.

### 2.6. Pharmacophore Modeling

The structure-based pharmacophore model was generated using the LigandScout software package [31], which uses an algorithm that studies and interprets ligand-receptor interactions such as charge transfer, hydrogen bonds, and hydrophobic regions of their macromolecular environment from PDB files, allowing the automatic building of the pharmacophore model. The generated pharmacophore model included the excluded volume spheres, which represents the inaccessible areas along any potential ligand [32].

## 3. Result and Discussion

Out of 25 constituents on which docking was performed, only 15 constituents passed the Lipinski rule of five (see Table 1 in Supplementary Material available online at http://dx.doi.org/10.1155/2014/903246). Docking results obtained for each ligand with the receptors were analyzed apart from docking energy, and binding modes and interaction of each ligand with the functional residues of IR (PDB ID: 1IR3), AR (PDB ID: 1USO), PTP1B (PDB ID: 2F70), and DPP-IV (PDB ID: 3F8S) were analyzed in detail by visually inspecting the docked complexes using MVD. The hydrogen bonds involved with bond length were also considered in docking results. Docking result of our constituents against each PDB structure is given in Supplementary Tables 2–5. On comparing the value of logP with docking score on different PDB, it is concluded that there is no relation between logP and docking score. Constituents having high logP value did not show good docking score on any PDB and constituents having intermediate logP value showed good docking score. H-bond acceptor can be seen as a tie-in with a docking score of constituents. The constituents having intermediate value of H-bond acceptor with intermediate value of logP may be considered as good antidiabetic constituents. The constituents having high value of H-bond acceptors also did not show good activity.

### 3.1. Insulin Receptor (IR)

The internal ligand ANP showed MolDock score value −268.525 on PDB ID: 1IR3, while three constituents, namely, secoisoresinol, pinoresinol, and cedeodarin showed maximum value of −123.346, −122.854, and −173.749, respectively. Amino acid residues, namely, Ser 1006, Lys 1030, Asp 1083, Met 1079, and Glu 1077, were the main amino acid residues involved in the interaction of internal ligand and most of the active constituents in 1IR3.

### 3.2. Aldose Reductase (AR)

MolDock score value of internal ligand IDD594 was −156.549, while pinoresinol constituents, namely, secoisoresinol, pinoresinol, and cedeodarin, showed maximum value of −173.78, −146.801, and −198.027, respectively. Amino acid residues, namely, Tyr 48, Ser 210, and Tyr 209, were having interaction with internal ligand, whereas constituents mainly interact with Trp 111, His 110, Asn 160, Cys 298, Asp 216, Leu 212, Asp 43, Thr, 199, and Trp 20 of the said receptor.

### 3.3. Dipeptidyl Peptidase-IV (DPP-IV)

In case of PDB ID: 2F70 internal ligand UN608 showed MolDock score value-70.6666 while three constituents, namely, caffeic acid, kaempferol, and cedeodarin showed maximum value of 101.897, −104.744, and −95.3797, respectively. Amino acid residues of PDB ID: 2F70 involved interactions with the internal ligand and constituents were Arg 221, Ser 216, Gly 218, Ile 219, Gly 220, Asp, 181, Gly 86, and His 214.

### 3.4. Protein Tyrosine Phosphatase 1-Beta (PTP-1*β*)

The internal ligand PF2 showed MolDock score value of −107.94 on PDB ID: 3F8S, while three constituents, namely, catechin, secoisoresinol, and pinoresinol showed maximum value of −112.972, −106.909, and −114.634, respectively. Constituents showed the least interaction with this receptor. Still Asn 710, Tyr 662, and Ser 630 were key amino acid residues for forming hydrogen bonds.

**Figure 1:**
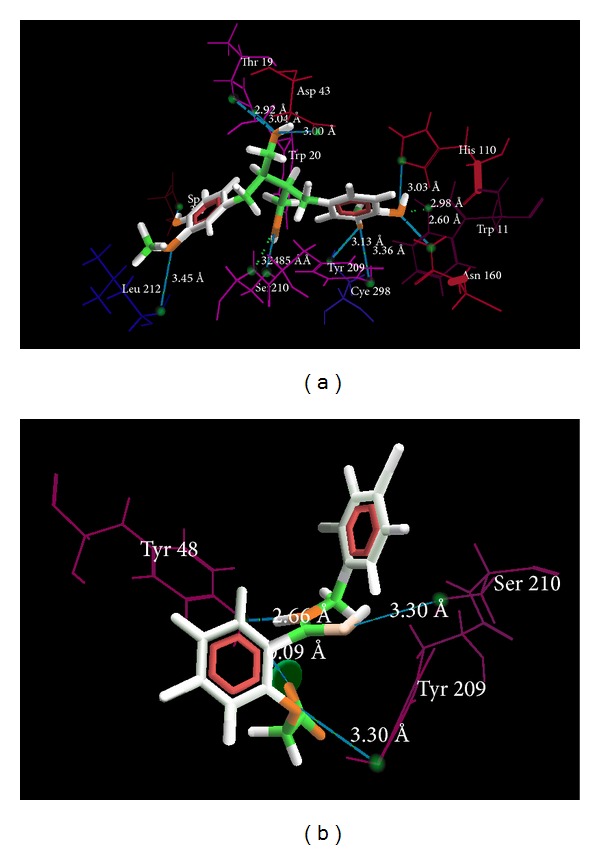
(a) Binding mode of secoisoresinol (green) into the binding site of aldose reductase receptor (PDB ID: 1US0). (b) Binding mode of IDD594 (green) into the binding site of aldose reductase receptor (PDB ID: 1US0).

From the results of docking score values on different receptors for antidiabetic activity, it is observed that constituents, namely, secoisoresinol, pinoresinol, and cedeodarin showed the best docking results on almost all the receptors, while most significant effects were observed on PDB ID: 1US0 against internal standard IDD594. The interaction of the standard and secoisoresinol is given in Figures 1(a)-1(b).

**Figure 2:**
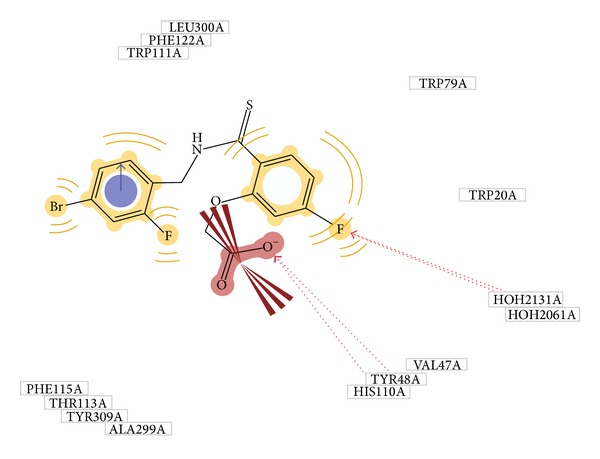
Pharmacophore model of 1US0.

The traditional medicinal system has plenty of opportunities, which are however needed to be explored till date for the treatment of many ailments [33]; if one can employ the modern computational chemistry tools for exploring the potential of the traditional medicinal system, then astonishing results can be received. Similar studies have been taken away in the past by many scientists where bioactive compounds are docked on particular receptor to evaluate its affinity [34–36]. In our work, we used two approaches of structure based drug designing, namely, molecular docking and pharmacophore modeling for measuring the potential antidiabetic components and their mechanism of activity. The primary aim of selecting the four different receptors was to distinguish the major pathway through which* Pinus roxburghii* exhibits its antidiabetic potential. From our docking results we found that it was aldose reductase on which active constituents from* Pinus roxburghii* were found to be most active. The role of aldose reductase inhibitors in diabetes has been corroborated by many researchers [37]. Further, our docking on the enzyme 1US0 (aldose reductase) revealed that secoisoresinol, pinoresinol, and cedeodarin have the highest affinity for AR. Our results were validated by generations of the pharmacophore model which predicts Tyr48 and His 110 as an indispensable essential for the formation of H-bonding with ligand (Figure 2). In our molecular docking simulation on 1US0, we found that internal ligand is interacting with Tyr 48 whereas secoisoresinol which has the highest MolDock score has interaction with His 110; this data is well correlated with the pharmacophore model.

## 4. Conclusion

17 constituents from* Pinus roxburghii* were docked on different receptors out of which secoisoresinol, pinoresinol, and cedeodarin showed the highest affinity for the AR. Pharmacophore model developed with the help of LigandScout predicts that Tyr 48 and His 110 are needed for the formation of H-bonding with ligand. Secoisoresinol which has highest MolDock score showed interaction with His 110. Moreover, antidiabetic effect of secoisoresinol has been shown in animal model also [38]. This clearly indicated that secoisoresinol from* Pinus roxburghii* can be utilized to care for diabetes. Our studies may lay the base of further exploration of the* Pinus roxburghii* for its antidiabetic potential. The above findings also validate the ethnopharmacological knowledge on this plant. Hence, it can be concluded that* Pinus roxburghii *has high potential as antidiabetic especially against aldose reductase pathway in diabetes.

## Supplementary Material

Supplementary Table 1 provides a list of chemical constituents reported in *Pinus roxburghii*. Supplementary Tables 2-5 provide information about docking scores, amino acid residue, atom of the ligand, H-boond length (A^0^) of chemical constituents from *Pinus roxburghii* on different receptors having PDB ID: (1IR3, 1US0, 2f70, 3F8S).

## Figures and Tables

**Table 1:** 2D structures of 25 chemical constituents from *Pinus roxburghii*.

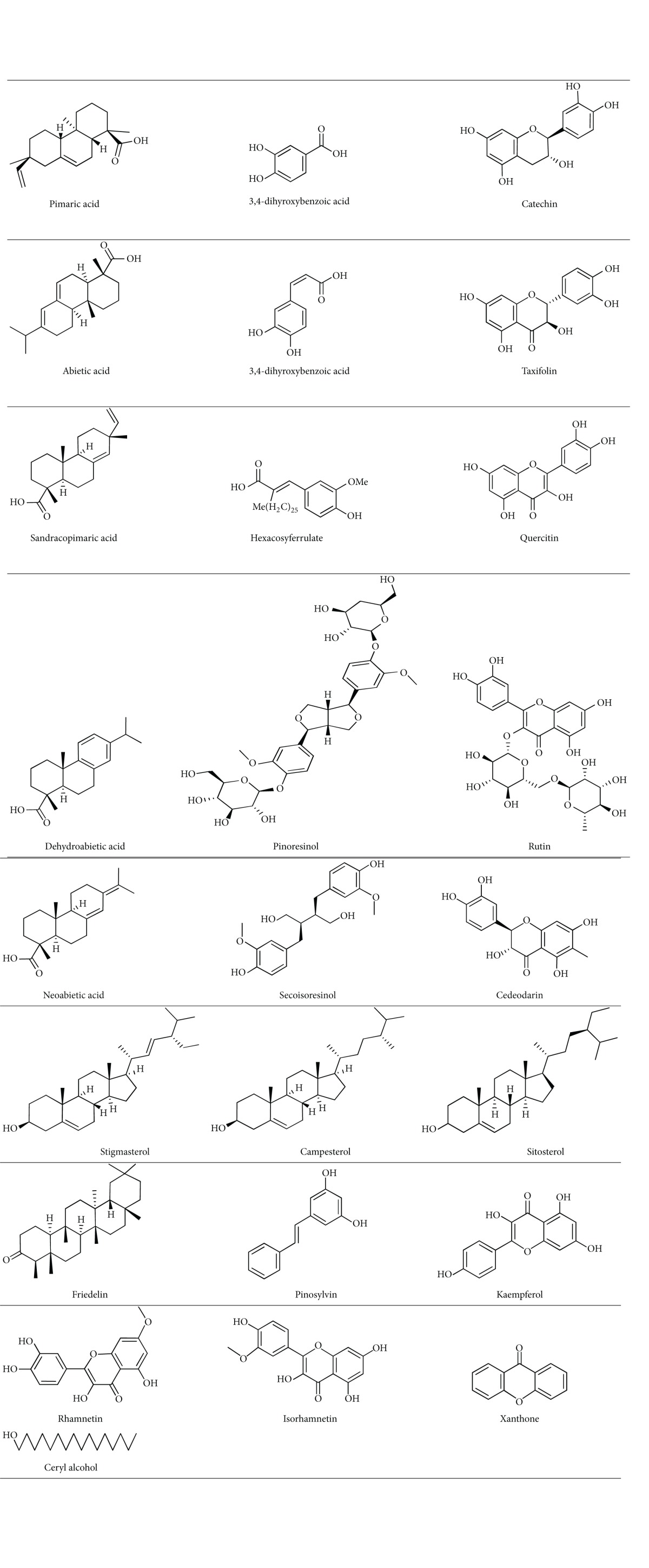

**Table 2:** MolDock score of internal ligand and extracted internal ligand of docked receptor-ligand complex structures.

PDB I.D	Internal ligand	MolDock score (internal ligand)	MolDock score (extracted internal ligand)
1IR3	ANP	−268.525	−268.525
1US0	IDD594	−155.639	−155.639
2F70	UN608	−70.6666	−70.6666
3F8S	PF2	−107.94	−107.94
