# Supplementary material for: Pharmacophore Modeling and Molecular Docking Studies on Pinus roxburghii as a Target for Diabetes Mellitus
Source: Adv Bioinformatics. 2014 Jul 10;2014:903246. doi: 10.1155/2014/903246 (PMC4120483; doi:10.1155/2014/903246)
Supplement: Supplementary file 1 — Supplementary Table 1 provides a list of chemical constituents reported in Pinus roxburghii. Supplementary Tables 2-5 provide information about docking scores, amino acid residue, atom of the ligand, H-boond length (A0) of chemical constituents from Pinus roxburghii on different receptors having PDB ID: (1IR3, 1US0, 2f70, 3F8S). [file 903246.f1.pdf]

Table 1 List of chemical constituents reported in *Pinus roxburghii*

S.No	Chemical constituent	CHEMSPIDER ID	LOG P	H BOND Acceptor	H BOND donor	Mol wt	No of violation	TPSA
	**Resin acid**							
1.	Pimaric acid	16735968	4.957	2	1	302.458	0	37.299
2.	Abietic acid	10127	5.007	2	1	302.458	1	37.299
3.	Sandracopimaric acid	192262	4.957	2	1	302.458	0	37.299
4.	Dehydroabietic acid	85184	5.67	2	1	300.442	1	37.299
5.	Neoabietic acid	191824	5.312	2	1	302.458	1	37.299
	**Sterols**							
6.	Campesterol	151215	8.305	1	1	400.691	1	20.228
7.	Sitosterol	192962	8.62	1	1	414.718	1	20.228
8.	Stigmasterol	4444352	7.869	1	1	412.702	1	20.228
	**Simple acid**							
9.	3,4 dihyroxybenzoic acid	71	0.88	4	3	154.121	0	77.755
10.	3,4 dihyroxycinnamic acid	1266077	0.941	4	3	180.159	0	
	**Ferulates**							
11.	Hexacosyferrulate	------------	10.107	3	1	558.932	2	46.533
	**Lignans**							
12.	Pinoresinol	204822	2.587	6	2	358.39	0	77.392
13.	secoisoresinol	58845	2.075	6	4	362.422	0	99.38
	**Stilbens**							
14.	Pinosylvin	11286912	3.437	2	2	226.275	0	40.456
	**Flavonoids**							
15.	Catechin	8711	1.369	6	5	290.271	0	110.374
16.	Taxifoiln	621332	0.712	7	5	304.251	0	127.445
17.	Quercitin	4444051	1.683	7	5	302.238	0	131.351
18.	Rutin	4444362	-1.063	16	10	610.521	3	269.427
19.	Cedeodarin	158323	1.349	7	5	318.281	0	127.445
20.	Kaempferol	4444395	2.172	6	4	286.239	0	111.123
21.	Rhamnetin	4445008	2.219	7	4	316.265	0	120.357
22.	Isorhamnetin	4444973	1.99	7	4	316.265	0	120.357
23.	Xanthone	6753	3.57	2	0	196.205	0	30.211
	**Triterpenoid**							
24.	Friedelin	82597	7.854	1	0	426.729	1	17.071
	**Fatty alcohol**							
25.	Ceryl alcohol	61478	9.655	1	1	382.717	1	20.228

Table 2 Docking scores of Chemical constituents from *Pinus roxburghii* using PDB ID: 1IR3

**S.no**	**Compound**	**MolDock Score**	**Amino acid residue**	**Atom of the ligand**	**H-boond length (A^0)**
**1.**	**ANP**	**-268.525**	**Ser 1006**<br>**Lys 1030**<br><br>**Asp 1083**<br>**Ser 1006**<br>**Met 1079**<br><br>**Glu 1077**	**O of SO_2**<br>**3-OH of**<br>**furan**<br>**N of NH_2**<br>**O of SO_2**<br>**O of**<br>**Pyranose**<br>**O of 4-OH**	**2.80**<br>**2.79**<br><br>**2.02**<br>**3.40**<br><br>**3.10**<br>**3.07**
2.	3,4 dihyroxybenzoic acid	-82.7528	Ser 1006<br>Ser1006<br>Gln 1004	O of CO2<br>O of CO2<br>O of ligand	2.82<br>3.12<br>2.99
3.	Catechin	-90.1103	Met 1079<br>Met 1079<br>Asp 1083<br>Lys 1030	O of 4-OH<br>O of 4-OH<br>O of 4-OH<br>O of OH	3.14<br>3.32<br>2.99<br>3.23
**4.**	**Secoisoresinol**	**-123.346**	**Glu 1077**<br>**Met 1079**<br>**Asp1083**<br>**Lys 1030**	**O of 4-OH**<br>**O of 4-OH**<br>**O of -OH**<br>**O of 4-OH**	**3.29**<br>**3.04**<br>**2.97**<br>**3.12**
5.	Sandracopimaric acid	-104.622	Lys 1030	O of -CO	3.03
**6.**	**Pinoresinol**	**-122.854**	**Met 1079**<br>**Met 1079**	**O of 4-OH**<br>**O of 4-OH**	**3.12**<br>**2.91**
7.	Taxifoiln	-96.0355	Leu 1002<br>Asp 1083<br>Asp 1083<br>Lys 1030<br>Lys 1030	O of 4-OH<br>O of 3-OH<br>O of 3-OH<br>O of –OH<br>O of 4-OH	3.12<br>2.37<br>2.64<br>3.10<br>2.50
8.	Caffeic acid	-98.7362	Lys 1030<br>Lys 1030	O of 3-OH<br>O of 4-OH	2.60<br>2.75
9.	Quercitin	-95.4304	Leu 1002<br>Gln 1004<br>Met 1079<br>Met 1079<br>Met 1079	O of 4-OH<br>O of 4-OH<br>O of 2-OH<br>O of 2-OH<br>O of 4-OH	2.94<br>2.85<br>2.56<br>2.60<br>3.23
10.	Kaempferol	-97.722	Leu 1002<br>Lys 1030<br><br>Lys 1030	O of 4-OH<br>O of 5-OH of<br>quinoline<br>O of 5-OH of	3.39<br>3.11<br><br>2.67

				quinoline	
11.	Isorhamnetin	-101.084	Lys 1030<br><br>Lys 1030<br><br>Leu 1002	O of 4-OH of quinoline<br>O of 5-OH of quinoline<br>O of 4-OH	2.72<br><br>3.13<br><br>3.13
12.	Rhamnetin	-97.8341	Lys 1030<br>Lys 1030<br>Leu 1002	O of 4-OH<br>O of 5-OH<br>O of 4-OH	2.65<br>3.13<br>3.10
13.	Pinosylvin	-90.5397	Lys 1030<br>Ser 1006	O of 3-OH<br>O of 5-OH	2.88<br>3.37
14.	Pimaric acid	-96.3319	No interaction		
15.	Hexacosyferrulate	-5.52251	No interaction		
16.	Xanthone	-70.433	Met1079	4-OH pyranose	2.82
17.	Cedeodarin	-173.749	No interaction		

Table 3 Docking scores of Chemical constituents from *Pinus roxburghii* using PDB ID: 1USO

**S.no**	**Compound**	**MolDock Score**	**Amino acid residue**	**Atom of the ligand**	**H-boond length (A^0)**
1.	IDD594	-155.639	Tyr48<br>Tyr48<br>Tyr209<br>Ser210	O of COOH<br>N of C=N<br>O of 2-OCH_2<br>S of SH	3.09<br>2.66<br>3.41<br>3.30
2.	3,4 dihyroxybenzoic acid	-95.9973	Ser 214<br>Leu 212<br>Lys 262<br>Ile 260	O of C=O<br>O of C=O<br>O of C=O<br>O of -OH	3.14<br>3.10<br>3.10<br>2.83
3.	Catechin	-134.751	Cys 303<br>Tyr 309<br>Ala 299<br>Cys 298<br>Tyr 209<br>His 110<br>Tyr 309	O of -OH<br>O of -OH<br>O of -OH<br>O of -OH<br>O of -OH<br>O of -OH<br>O of -OH	3.02<br>2.51<br>3.10<br>2.60<br>3.15<br>2.79<br>3.44

4.	Secoisoresinol	-180.947	Trp111<br>His110<br>Asn160<br>Cys298<br>Tyr48<br>Ser210<br>Ser210<br>Asp216<br>Leu212<br>Asp43<br>Thr19<br>Trp20	O of -4OH<br>O of -4OH<br>O of -4OH<br>O of -4OH<br>O of -4OH<br>O of –OH<br>O of –OH<br>O of -4OH<br>O of 3-OCH_3<br>O of -OH<br>O of –OH<br>O of -OH	2.98<br>3.03<br>2.60<br>3.13<br>3.36<br>2.65<br>3.48<br>3.25<br>3.12<br>3.10<br>2.92<br>3.04
5.	Sandracopimaric acid	-125.402	Tyr 48	O of -OH	2.60
6.	Pinoresinol	-146.801	Ser 210<br>Ser 159<br>Gln 183<br>Pro 215	O of -O-<br>O of -OH<br>O of -OH<br>O of -4-OH	2.78<br>3.52<br>2.83<br>2.38
7.	Taxifoiln	-140.194	Asp 43<br>Gln 183<br>Lys 77<br>Cys 298<br>Tyr 209<br>Ser 210<br>Ile 260<br>Leu 212<br>Pro 211<br>Ser 210	O of -OH<br>O of -OH<br>O of -OH<br>O of -OH<br>O of -OH<br>O of Pyranose<br>O of -OH<br>O of -OH<br>O of -OH<br>O of Pyranose	3.04<br>2.63<br>3.27<br>2.80<br>3.47<br>2.92<br>2.99<br>2.74<br>3.15<br>3.55
8.	Caffeic acid	-115.197	Ser 214<br>Leu 212<br>Lys 262<br>Ile 260<br>Ser 210<br>Asp 43	O of C=O<br>O of C=O<br>O of C=O<br>O of -OH<br>O of -OH<br>O of -OH	3.04<br>3.02<br>3.00<br>3.03<br>3.00<br>3.49
9.	Quercitin	-141.885.067	Ile 260<br><br>Ser 210<br><br>Tyr 209<br>Cys 298<br>Lys 77<br>Gln 183<br>Asp 43<br>Pro 211<br>Leu 212	N of Quinoloine<br>N of Quinoloine<br>O of -OH<br>O of -OH<br>O of -OH<br>O of -OH<br>O of -OH<br>O of -OH<br>O of -OH	2.62<br><br>3.01<br><br>3.42<br>2.82<br>3.10<br>2.70<br>2.88<br>3.26<br>2.73

10.	Kaempferol	-138.548	Ile 260<br>Asp 43<br>Lys 77<br>Ser 210<br><br>Ser 210<br><br>Cys 298<br>Tyr 209<br>Gln 183	O of -OH<br>O of -OH<br>O of -OH<br>N of<br>Quinoline<br>N of<br>Quinoline<br>O of -OH<br>O of -OH<br>O of -OH	2.59<br>3.10<br>3.20<br>2.94<br><br>3.55<br><br>2.87<br>3.48<br>2.93
11.	Isorhamnetin	-145.042	Lys 77<br>Gln 183<br>Asp 43<br>Tyr 209<br>Cys 298<br>Ser 210<br>Ile 260<br>Leu 212	O of -OH<br>O of -OH<br>O of -OH<br>O of -OH<br>O of -OH<br>N of<br>Quinoloine<br>O of -OH<br>O of -OCH_3	3.07<br>2.61<br>2.84<br>3.33<br>2.84<br>2.99<br>2.77<br>3.17
12.	Rhamnetin	-150.026	Asp 43<br>Lys 77<br>Gln 183<br>Cys 298<br>Asp 216<br>Tyr 209<br>Ser 210<br>Ser 210<br>Ile 260	O of 3-OH<br>O of 5-OH<br>O of 5-OH<br>O of 5-OH<br>O of 3-OH<br>O of 3-OH<br>O of 8-OH<br>N of<br>Quinoloine<br>O of –OH	3.00<br>3.10<br>2.84<br>2.95<br>2.51<br>3.56<br>3.53<br>3.03<br>2.48
13.	Pinosylvin	-134.563	Asp 43<br>Tyr 48<br>Asp 43<br>Thr 19<br>Trp 20	O of -OH<br>O of -OH<br>O of -OH<br>O of -OH<br>O of –OH	3.46<br>3.10<br>3.10<br>2.60<br>3.05
14.	Pimaric acid	-132.033	Tyr 48	O of -C=O	2.60
15.	Hexacosyferrulate	-190.717	Tyr 309<br>Cys 303	O of -C=O<br>O of -C=O	3.34<br>3.06
16.	Xanthone	-109.842	Ser210<br>Trp20<br>Lys21	O of pyranose<br>4-OH<br>pyranose<br>4-OH<br>pyranose	2.96<br>2.88<br>2.50
17.	Cedeodarin	-198.027	Asp43<br>Lys262<br>Ser214<br>Leu212	N of NH<br>O of CO_2<br>O of CO_2<br>O of CO_2	2.90<br>2.75<br>3.43<br>3.25

			Asp216<br>Ser214<br>Ser210	N of NH_2<br>N of NH_2<br>N of CONH	3.54<br>3.38<br>3.22

Table 4 Docking scores of Chemical constituents from *Pinus roxburghii* using PDB ID: 2f70

**S.no**	**Compound**	**MolDock Score**	**Amino acid residue**	**Atom of the ligand**	**H-boond length (A^0)**
**1.**	**UN608**	**-70.6666**	**Arg 221**<br>**Arg 221**<br>**Ser 216**<br>**Gly 218**<br>**Ser 216**<br>**Ile 219**<br>**Gly 220**<br>**Asp 181**<br>**Arg 221**<br>**Gly 86**<br>**His 214**	**-O of SO_2**<br>**-O of SO_2**<br>**-O of SO_2**<br>**-O of SO_2**<br>**-O of SO_2**<br>**-O of SO_2**<br>**-O of SO_2**<br>**-O of Ether**<br>**-O of SO_2**<br>**-O of SO_2**<br>**-O of SO_2**	**2.85**<br>**2.65**<br>**2.65**<br>**3.10**<br>**3.08**<br>**2.89**<br>**1.94**<br>**3.22**<br>**2.60**<br>**2.79**<br>**2.88**
2.	3,4 dihyroxybenzoic acid	-93.2968	Ile 219<br>Gly 220<br>Gly 218<br>Arg 221<br>Arg 221<br>Tyr 46<br>Cys 215	O of C=O<br>O of C=O<br>O of C=O<br>O of C=O<br>O of C=O<br>O of 3-OH<br>O of C=O	2.94<br>2.60<br>3.27<br>3.11<br>3.10<br>2.91<br>2.60
3.	Catechin	-83.535	Arg 221<br>Gly 220<br>Tyr 46<br>Arg 221	O of 4-OH<br>O of 5-OH<br>O of pyranose<br>O of 4-OH	3.08<br>2.95<br>3.17<br>3.27

<table>
<tr><td>4.</td><td>Secoisoresinol</td><td>19.1388</td><td>Gln 218
Gly 220
Cys 215
Ile 219
Arg 221
Arg 221
Arg 221
Gly 259</td><td>O of 3 -OH
O of 4 OH
O of CH_3
O of 4OH
O of OCH_3
O of OCH_3
O of OCH_3
O of 4OH</td><td>3.15
2.55
2.99
2.66
3.29
2.46
3.20
3.57</td></tr>
<tr><td>5.</td><td>Sandracopimaric acid</td><td>-87.863</td><td>Ile 219
Gly 218
Ala 217
Arg 221
Arg 221</td><td>O of C=O
O of C=O
O of C=O
O of C=O
O of C=O</td><td>3.28
2.84
3.01
2.76
3.10</td></tr>
<tr><td>6.</td><td>Pinoresinol</td><td>-84.4847</td><td>Arg 221
Ser 216
Ala 217
Cys 215</td><td>O of 4-OH
O of 4-OH
O of OCH_3
O of OCH_3</td><td>2.91
2.95
3.51
3.40</td></tr>
<tr><td>7.</td><td>Taxifoiln</td><td>-88.3651</td><td>Arg 221
Asp 181
Arg 221
Ser 216
Cys 215
Gly 220
Arg 221
Asp 48
Ile 219</td><td>O of 4-OH
O of 4-OH
O of 3-OH
O of 4-OH
O of 4-OH
O of 3-OH
O of 4-OH
O of 3-OH
O of 3-OH</td><td>2.99
3.46
3.48
3.91
3.26
2.42
2.60
3.41
3.29</td></tr>
<tr><td>8.</td><td>Caffeic acid</td><td>-101.897</td><td>Tyr 46
Gly 2220
Ile 219
Arg 221
Arg 221
Ser 216
Cys 215
Arg 221
Cys 215</td><td>3-OH
O of 3-OH
C=O
O of 4 -OH
O of 4
O of 4
O of 4
O of 3-OH
O of 3-OH</td><td>3.20
2.59
3.28
2.67
3.39
3.40
3.30
3.34
2.46</td></tr>
</table>

9.	Quercitin	49.6707	Tyr 46<br>Arg 221<br>Gly 262<br>Arg 221<br>Ser 216<br>Tyr 46<br>Pro 180<br>Trp 179<br>Asp 181	O of -OH<br>O of -OH<br>O of -OH<br>O of -OH<br>O of -OH<br>O of -OH<br>O of -OH<br>O of -OH<br>O of -OH	3.17<br>3.01<br>2.93<br>3.39<br>3.51<br>3.26<br>310<br>2.96<br>2.74
**10.**	**Kaempferol**	**-104.744**	**Try 46**<br>**Asp 181**<br>**Arg 221**<br>**Gly 220**<br>**Cys 215**	**4-OH of Quinoline**<br>**5-OH of Quinoline**<br>**7-OH of Quinoline**<br>**7-OH of Quinoline**<br>**7-OH of Quinoline**	**3.04**<br>**2.95**<br>**3.07**<br>**2.80**<br>**2.61**
11.	Isorhamnetin	-89.1876	Arg 221<br>Ser 216<br>Gly 220<br>Asp 181<br>Asp 48	3-OH<br>3-OH<br>4-OH<br>3-OH<br>3-OH of Quinoline	2.94<br>3.48<br>3.22<br>3.10<br>3.08
12.	Rhamnetin	17.7531	Asp181<br>Lys 120<br>Tyr 46<br>Arg 221<br>Met 258	3-OH of Quinoline<br>3-OH of OCH_3<br>3-OH of Quinoline<br>O of OCH_3<br>O of 4-OH	3.18<br>4.95<br>3.47<br>3.06<br>3.10
13.	Pinosylvin	-88.6779	Arg 221<br>Arg 221<br>Ser 216<br>Gly 220<br>Cys 215	O of OH<br>O of OH<br>O of OH<br>O of -OH<br>-OH	3.16<br>2.57<br>2.60<br>2.61<br>3.09
14.	Pimaric acid	-68.8936	No interaction		
15.	Hexacosyferrulate	291.3442	Ser 270	O of –OCH_3	2.66
16.	Xanthone	-83.1765	Gln262	4-OH pyranose	3.18
17.	Cedeodarin	95.3797	Lys116	O of CO_2	2.78

			Gln262<br>Gly220	O of CO_2<br>N of CONH	2.73<br>3.34

Table 5 Docking scores of Chemical constituents from *Pinus roxburghii* using PDB ID: 3F8S

**S.no**	**Compound**	**MolDock Score**	**Amino acid residue**	**Atom of the ligand**	**H-boond length (A^0)**
**1.**	**Pf2**	**-107.94**	**Asn 710**<br>**Tyr 662**<br>**Ser 630**<br>**Ser 630**	**O of –OH**<br>**O of Furan**<br>**O of Furan**<br>**O of Furan**	**3.31**<br>**3.50**<br>**3.17**<br>**3.40**
2.	3,4 dihyroxybenzoic acid	-71.4059	Arg 125<br>Arg 125<br>Asp 709<br>Lys 122<br>Asp 793	O of C=O<br>O of C=O<br>O of OH<br>O of OH<br>O of OH	3.06<br>3.06<br>2.60<br>2.93<br>3.00
3.	Catechin	-112.972	Tyr 585<br>Glu 361<br>Ile 407<br>Glu 361<br>Arg 358<br>Arg 356<br>Arg 356	O of –OH<br>O of –OH<br>O of -OH<br>O of -OH<br>O of –Oh<br>O of -OH<br>O of Pyranose	3.17<br>3.09<br>3.18<br>3.10<br>3.48<br>3.03<br>2.85
4.	Secoisoresinol	-106.909	Tyr 585<br>Arg 429<br>Arg 358<br>Ile 405	OCH3<br>OCH3<br>O of OH<br>O of OH	3.10<br>3.47<br>2.60<br>3.08
5.	Sandracopimaric acid	-98.5671	No interaction		
6.	Pinoresinol	-114.634	Phe 357<br>Arg 358<br>Arg 358<br>Arg 358<br>Trp 215	3 –OH<br>O og OCH_3<br>O of Furan<br>O of Furan<br>O of OH	3.10<br>3.54<br>3.06<br>3.22<br>3.51
7.	Taxifoiln	-91.4079	Asp 739<br>Glu 205<br>Asp 739<br>Lys 122	O of -OH<br>O of -OH<br>O of -OH<br>O of -OH	3.10<br>2.91<br>3.10<br>2.81

8.	Caffeic acid	-86.569	Asp 709<br>Asp 709<br>Arg 125	O of –OH<br>O of -OH<br>O of –C=O	2.87<br>2.76<br>2.60
9.	Quercitin	-88.291	Arg 358<br>Arg 358<br>Glu 361<br>Pro 359<br>Asp 302<br>Val 303	N of quinoline<br>N of quinoline<br>O of 4 OH quinoline<br>O of 4 OH quinoline<br>O of 3OH quinoline<br>O of 3 OH	3.12<br>2.78<br>2.81<br>2.60<br>2.98<br>2.94
10.	Kaempferol	-99.4805	Asp 739<br>Asp 739<br>Lys 122<br>Glu 205<br>Glu 205	4 OH of quinoline<br>5 OH of quinoline<br>5 OH of quinoline<br>7 OH of quinoline<br>7 OH of quinoline	2.85<br>3.10<br>2.77<br>2.93<br>2.93
11.	Isorhamnetin	-97.2701	Glu 408<br>Ile 407<br>Ile 407<br>Ile 407<br>Arg 356<br>Arg 356<br>Arg 382	4 OH quinoline<br>3 OH quinoline<br>3 OH quinoline<br>4 OH quinoline<br>O of $–OCH_3$<br>O of $–OCH_3$<br>O of -OH	2.96<br>2.90<br>3.37<br>3.54<br>3.09<br>2.73<br>3.38
12.	Rhamnetin	-96.9684	Arg 358<br>Arg 356<br>Arg 356<br>Arg 382<br>Ile 407<br>Ile 407<br>Ile 407<br>Ile 407<br>Glu 408	O of -OH<br>O of -OH<br>O of -OH<br>O of -OH<br>O of –OH<br>O of –OH<br>O of –OH<br>O of –OH<br>O of -OH	2.53<br>2.97<br>2.76<br>3.18<br>3.49<br>3.00<br>3.49<br>2.60<br>3.14
13.	Pinosylvin	-97.9143	Ser 630<br>His 740	O of -OH	2.87<br>3.31

				O of -OH	
14.	Pimaric acid	-91.6603	Arg 356<br>Arg 356	O of C=O<br>O of C=O	3.03<br>3.23
15.	Hexacosyferrulate	-59.1203	Arg 356<br>Arg 356	O of C=O<br>O of C=O	3.12<br>2.60
16.	Xanthone	-81.6503	Phe713<br>Thr706<br>Ser239<br>Tyr241	O of 4-OH pyranose<br>O of 4-OH pyranose<br>O of pyranose<br>O of pyranose	3.29<br>2.60<br>2.60<br>2.75
17.	Cedeodarin	-100.132	Thr736<br>Thr706<br>Tyr241<br>Ser239<br>Gln714<br>Phe713<br>Ser239<br>Thr706<br>Glu237	O of C=O<br>O of C=O<br>N of CONH<br>O of C=O<br>O of C=O<br>O of C=O<br>O of CONH<br>N of NH_2<br>O of 4-OH cyclohexane	3.01<br>3.13<br>3.02<br>3.40<br>3.10<br>3.53<br>3.17<br>3.09<br>3.36
